# Mechanisms underlying the activity-dependent regulation of locomotor network performance by the Na^+^ pump

**DOI:** 10.1038/srep16188

**Published:** 2015-11-06

**Authors:** Hong-Yan Zhang, Laurence Picton, Wen-Chang Li, Keith T. Sillar

**Affiliations:** 1 School of Psychology and Neuroscience, University of St Andrews, St Andrews KY16 9JP, United Kingdom

## Abstract

Activity-dependent modification of neural network output usually results from changes in neurotransmitter release and/or membrane conductance. In *Xenopus* frog tadpoles, spinal locomotor network output is adapted by an ultraslow afterhyperpolarization (usAHP) mediated by an increase in Na^+^ pump current. Here we systematically explore how the interval between two swimming episodes affects the second episode, which is shorter and slower than the first episode. We find the firing reliability of spinal rhythmic neurons to be lower in the second episode, except for excitatory descending interneurons (dINs). The sodium/proton antiporter, monensin, which potentiates Na^+^ pump function, induced similar effects to short inter-swim intervals. A usAHP induced by supra-threshold pulses reduced neuronal firing reliability during swimming. It also increased the threshold current for spiking and introduced a delay to the first spike in a train, without reducing subsequent firing frequency. This delay was abolished by ouabain or zero K^+^ saline, which eliminate the usAHP. We present evidence for an A-type K^+^ current in spinal CPG neurons which is inactivated by depolarization and de-inactivated by hyperpolarization, and accounts for the prolonged delay. We conclude that the usAHP attenuates neuronal responses to excitatory network inputs by both membrane hyperpolarization and enhanced de-inactivation of an A-current.

Mounting evidence suggests that the ubiquitously expressed Na^+^/K^+^ ATPase (aka the Na^+^ pump) plays important roles in regulating the output of neural networks in the CNS in health and in disease states (reviewed in ref. 1). Data obtained from studies across a range of species (from flies to mice), developmental stages (from embryos to adults) and functional brain areas (from sensory to motor systems) have revealed that Na^+^ pump function can be modified by neural network activity^2^,^3^,^4^,^5^,^6^,^7^,^8^. This is due to the fact that the Na^+^ pumps of constituent neurons sense and respond to the increase in intracellular Na^+^ ion concentration that accompanies intense action potential firing. In turn, the enhanced pump activity drives more Na^+^ out than K^+^ into the neuron in a 3:2 ratio, resulting in an outward pump current that shifts the membrane potential to a more hyperpolarized level. Thus the pumps function homeostatically as a negative feedback system to restore low intracellular sodium following intense activity. In effect, they act as spike rate monitors.

We have recently shown that in the spinal central pattern generator (CPG) network controlling swimming locomotion in young frog tadpoles, Na^+^ pumps provide a form of short term memory that links future to past locomotor CPG activity^9^. As a consequence of increased pump activity long, intense bouts of locomotion are followed by shorter, weaker ones if evoked within about a 1 minute time window. This period broadly matches the duration of a post-swim pump-mediated hyperpolarization, which we termed the ultraslow afterhyperpolarization (usAHP). However, the underlying mechanism and, in particular, how the usAHP actually causes reduced network output remain unknown.

Here we first document the detailed changes in swim parameters of a second episode and how these changes relate to the precise timing of swim bouts evoked after a well-rested first episode. Similar changes can be induced using the sodium ionophore, monensin, which increases intracellular Na^+^ and thus mimics intense neuronal firing^10^,^11^. We then examine the effects of the pump-based usAHP on the integrative electrical properties of identified CPG neurons. Besides increasing the excitation threshold, another dramatic effect of the usAHP is to introduce a delay to the first spike in a train so that the number of spikes for a given suprathreshold input is reduced. This delay displays many of the hallmarks of an A-type K^+^ current (*I*_A_) being brought into play by the usAHP via enhanced deinactivation at more negative membrane potentials. We provide voltage clamp and pharmacological evidence for the existence of an *I*_*A*_. Our data support the conclusion that the usAHP reduces locomotor network excitability by enhancing *I*_*A*_ and shifting the membrane potential away from the threshold for firing in a sub-set of CPG neurons.

## Results

### Short inter-swim interval affects subsequent network activity

**Figure 1:**
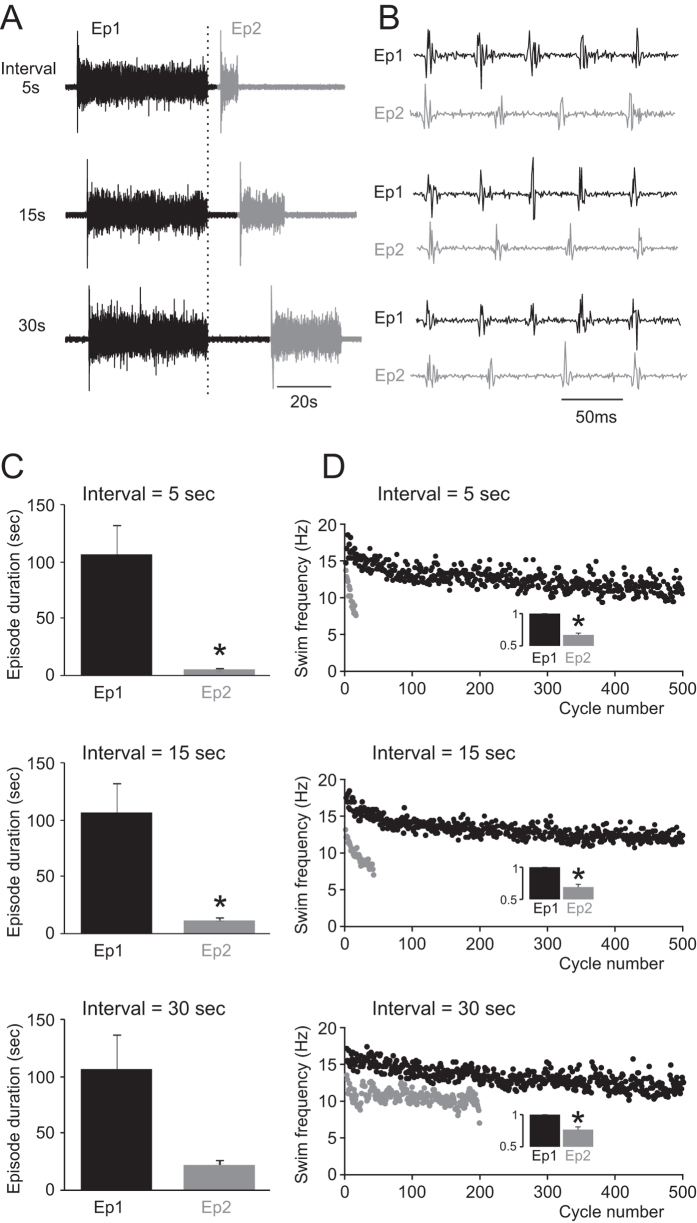
Swimming frequency and episode duration are affected by inter-swim interval. (**A**) Pairs of fictive swimming episodes induced by the same skin stimuli with inter-swim intervals set at 5, 15 and 30 seconds. (**B**) Swimming bursts at the beginning of the first (Ep1) and second (Ep2) episodes shown in (**A**) respectively. Note that the cycle periods of Ep2 are longer compared to Ep1. (**C**) Average episode duration of Ep2 was significantly shorter than Ep1 at the 5 and 15 second intervals (*P < 0.05, n = 5). (**D**) Time series measurements of swimming frequencies of Ep1 (black) and Ep2 (grey) for the three defined intervals. Insert bar graphs: average normalised swimming frequency of Ep1 and Ep2. Ep2 swimming frequency is lower than Ep1 (*P < 0.05, n = 5).

We previously described a broadly linear relationship between swimming episode duration and inter-swim interval in *Xenopus* frog tadpoles^9^, but the relationship was not explored systematically. Here we have used a more controlled double episode protocol in which two consecutive fictive swimming episodes with 5, 15 or 30 second intervals were induced by brief electrical stimulation of the skin. Pairs of episodes were separated by a rest period of at least two minutes to ensure that episode 1 was not affected by the usAHP and therefore episode 1 in each pair consistently had a long duration. When the interval was set to 5 or 15 seconds, episode 2 duration was significantly shorter than episode 1 (Fig. 1A,C; n = 5; P < 0.05). By 30 seconds, the effect on episode duration no longer reached significance (p = 0.091), suggesting that the usAHP, and its effect on swimming episode duration, had significantly diminished with a longer interval between episodes. The second episode in the pair also displayed markedly slower swimming frequency, which rapidly declined during swimming episodes (Fig. 1B,D). Swimming frequency in episode 2 was significantly lowered for all three inter-swim intervals: to 66.8 ± 2.9% of episode 1 for the 5 s interval (p < 0.05; Fig. 1D; top); 69.1 ± 4.5% for the 15s interval (p < 0.05; n = 5; Fig. 1D; middle); and 76.6 ± 4.2% for the 30s interval (p < 0.05; n = 5; Fig. 1D; bottom). These data suggest that the excitability of the swim CPG network in the second episode has been lowered in an interval-dependent manner.

### Effects of short inter-swim interval on neuron firing reliability

**Figure 2:**
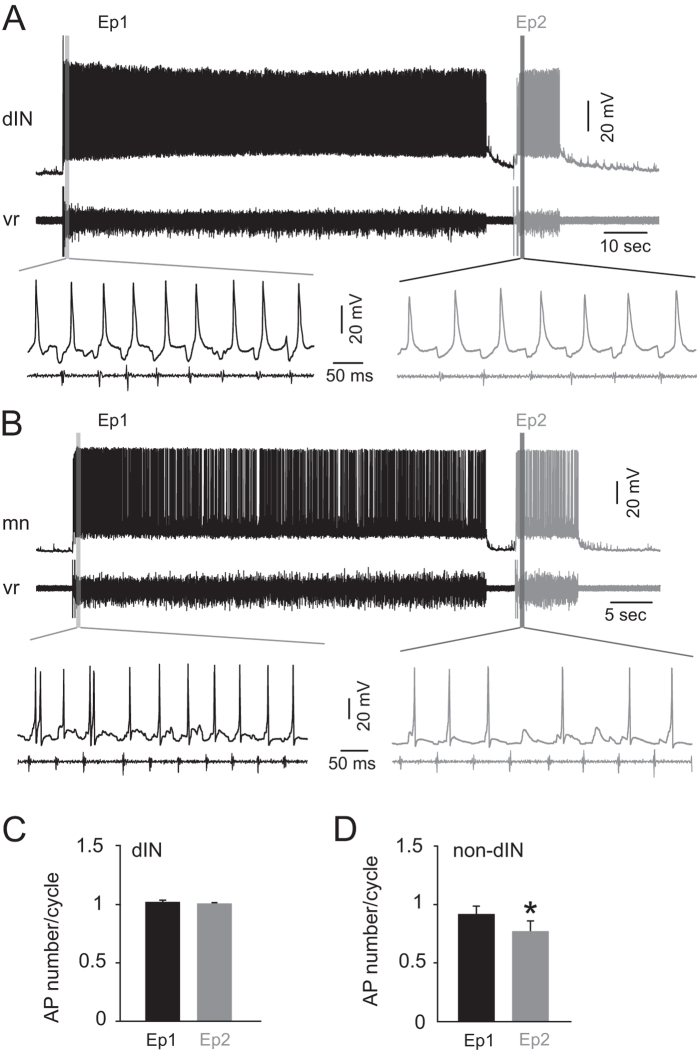
Short inter-swim interval has neuron type-specific effects on the firing reliability of CPG neurons. (**A**) Two swimming episodes with a short interval were induced by skin stimulation. A dIN was recorded simultaneously with ventral root (vr) activity. (**B**) MN firing during two swimming episodes with a short interval. Note that the firing reliability is lower during the second episode. (**C**) Mean dIN action potential (AP) number per swim cycle. The firing reliability of dINs is unaffected. (**D**) Mean non-dIN AP number per swim cycle. The firing reliability of non-dIN CPG neurons in the second episode was significantly reduced. (*P < 0.05, n = 17; paired *t*-test).

Short inter-swim intervals clearly weakened subsequent CPG network activity. Swimming frequency in *Xenopus* tadpoles is determined by the speed of dIN rebound firing, which in turn is affected by mid-cycle inhibition from cINs, as well as dIN tonic depolarization amplitude^12^. To determine how the swim network is affected by inter-swim interval, whole cell current clamp recordings were made from 26 rhythmically active neurons (10 motoneurons (MNs); 9 descending interneurons (dINs); 3 ascending interneurons (aINs); 1 commissural interneuron (cIN); 3 unidentified rhythmically active swim neurons). Pairs of swimming episodes with short intervals were induced using the same protocol as above (Fig. 2A,B) and the reliability of neuron firing in each episode was analysed and compared. In 9 dINs, no significant change of firing reliability was found; all dINs fired a single action potential per cycle in all cycles of all episodes recorded (16 trials in total; P = 0.44; Fig. 2A,C). As aINs, cINs and MNs fire less reliably during swimming compared to dINs, and all of them have a subset of neurons displaying usAHP^9^, we group these three types of neuron as non-dINs. In these 17 non-dINs (stated above) the firing reliability was significantly reduced in the second episode by an average of 15.9%, from 0.92 ± 0.07 to 0.77 ± 0.09 spikes per cycle (36 trials in total; paired *t*-test; P < 0.05; Fig. 2B,D). The firing reliability of non-dIN neurons was therefore affected by preceding network activity, but dIN firing during swimming was unaltered.

**Figure 3:**
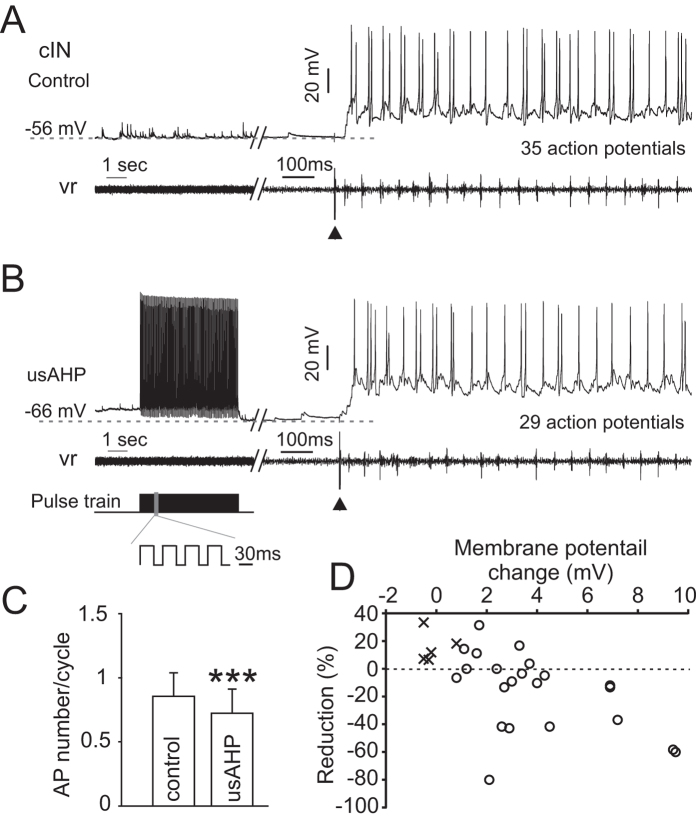
The usAHP reduces CPG neuron firing reliability. (**A**) Control firing of a non-dIN neuron and simultaneous ventral root activity; note the different time scales. (**B**) same neuron as in (**A**); a swimming episode was evoked immediately after a train of pulses had been used to induce repetitive firing and a usAHP. (**C**) Firing reliability of non-dINs in episodes initiated during a usAHP was lower compared to the control episodes (***P < 0.001, n = 11; paired *t*-test). (**D**) The reduction of neuron firing reliability has been plotted against the membrane potential change (potential before the suprathreshold pulse train – potential before swimming). Each trial from neurons displaying a usAHP (circle) and not displaying a usAHP (cross) are shown.

It has been shown that about 40% of non-dINs (but no dINs) display a usAHP (~1 min long) following repetitive firing, and it was assumed that the usAHP affects CPG neuron excitability in the subsequent swimming episode^9^. We next tested how the usAHP impacts upon neuronal excitability by injecting a train of 30 ms suprathreshold depolarizing pulses (20 ms interval, 5 seconds long, Fig. 3B) to mimic the excitation phase of each swimming cycle and to induce a usAHP in non-dINs. Shortly after this pulse train, a fictive swimming episode was evoked (Fig. 3B). The firing reliability of more than half of the non-dINs that displayed a usAHP (11 out of 17; 4 MNs; 3 cINs; 1 aIN; 3 unknown) was on average 15.3% lower than in control episodes (Fig. 3; paired *t*-test; P < 0.001; n = 11), indicating that the usAHP in individual neurons, rather than network activity during swimming, reduces the excitability of a subset of CPG neurons when responding to the same network drive. The firing reliability was not reduced during induced swimming in any of the non-dINs that did not display a usAHP (n = 3; Fig. 3D). These data indicate that the reduced firing reliability is directly linked to the membrane hyperpolarization associated with the usAHP.

### The usAHP alters CPG neuron electrical properties

**Figure 4:**
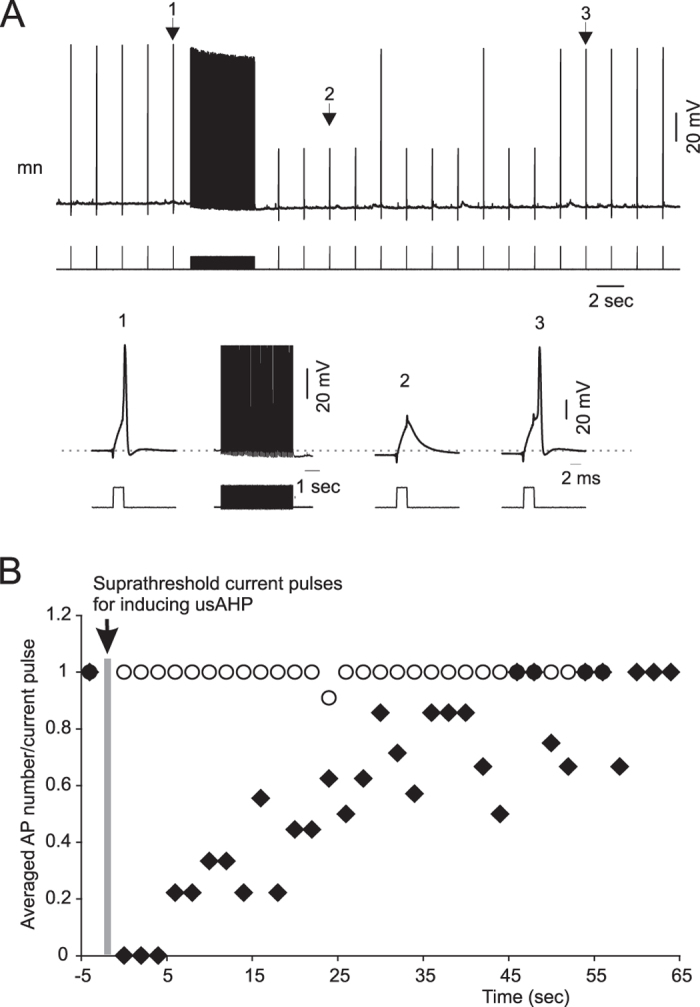
Current threshold for generating action potentials is increased during the usAHP. (**A**) Response of a MN to brief threshold current pulses (2 ms) before and during the usAHP. The suprathreshold current pulse train (30 ms pulse with 20 ms interval for 5 seconds) is used to trigger repetitive firing and a usAHP. (**B**) Pooled data of neuron responses to brief threshold current. Black diamonds: 9 non-dINs displayed a usAHP following suprathreshold current pulses (arrowed); open circles: 12 non-dINs without usAHP.

During swimming, all rhythmically active neurons receive excitatory drive from pre-synaptic dINs, whose firing is not altered during the usAHP period. However, non-dINs fire fewer action potentials during bouts of swimming initiated within the period of a usAHP, indicating that their response to the same excitatory synaptic inputs from dINs has been modified. To investigate how the electrical properties of these neurons have changed, trains of brief 2 ms depolarising pulses were applied to neurons before and during an induced usAHP to check if the threshold current (minimum current needed to evoke an action potential) remains the same (Fig. 4A). To induce a usAHP we injected either a 1 second long suprathreshold depolarizing pulse or a 5 seconds long 20Hz train of 30 ms depolarising pulses^9^. The 2 ms depolarizing pulse that successfully evoked action potentials before and after recovery from the usAHP, failed to do so during the usAHP (Fig. 4A,B; n = 9; 4 cINs; 2 MNs; 3 unidentified). When the same protocol was applied to neurons that did not display a usAHP, their threshold current was the same before and after repetitive firing (Fig. 4B; n = 12), indicating that the depolarization and repetitive firing caused by the long pulse used in the experiment did not affect the threshold current. Instead, it is the usAHP that directly affects neuron excitability.

**Figure 5:**
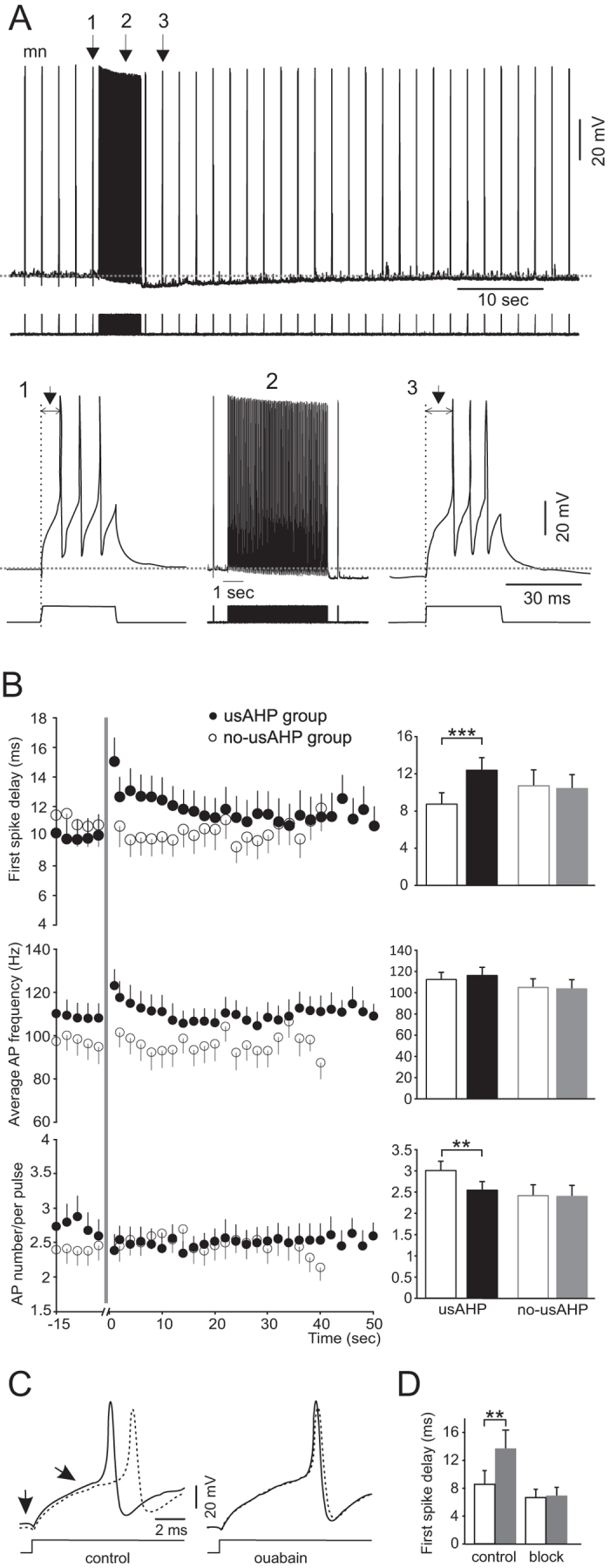
The effects of the usAHP on first spike delay, firing frequency and spike number. (**A**) 30 ms suprathreshold depolarizing current pulses were applied every 2 seconds before (1) and after (3) a train of 30 ms depolarizing pulses (2) that induces continuous firing on top of the depolarization. A usAHP (5.5 mV) appears following the 5 second pulse train (as in Fig. 4). The first spike delay (arrow) is longer in pulse (3) than (1). (**B**) Changes in mean first spike delay, firing frequency and AP number per pulse. The grey line represents the train of pulses in (**A**). Filled circle: neurons displaying a usAHP; open circle: neurons not displaying a usAHP. Bar graphs represent pooled data of different groups. Open bar: control period (before the grey line); closed bar: the first 5 seconds following the long pulse train (2 in **A**). Paired *t*-test: **P < 0.01; ***P < 0.001; n = 13. (**C**) Action potentials induced by a series of suprathreshold pulses. Left panel: control period; right panel: in the presence of ouabain; black trace: action potential induced by the first pulse; dashed trace: action potentials induced by the 5^th^ or 6^th^ pulse. Note the differences in resting membrane potential and first spike delay in the left panel (arrow), but not in the right panel. (**D**) Pooled data of control (left) and block (right) groups. Open bar: the first pulse in both groups; closed bar: the 5^th^ or 6^th^ pulse. The spike delays in control are different, but not after blocking the Na^+^ pump with ouabain or zero K^+^ saline (paired *t*-test: **P < 0.01, n = 6).

Multiple spikes can be induced in rhythmically active non-dIN swim neurons using longer, 30 ms suprathreshold depolarizing pulses, simulating the excitatory phase of the swimming cycle. When such pulses were applied before and during an induced usAHP (Fig. 5A), they were able to cause brief trains of action potentials. The average delay to the first spike of the train (duration from the start of the pulse to the spike peak; indicated by arrows), firing frequency and action potential number in the first 5 seconds of the usAHP period could then be compared to control. The first spike delay was significantly prolonged during the usAHP (Fig. 5A,B; paired *t*-test; n = 13; P < 0.001). Due to this prolonged delay, the number of spikes resulting from each 30 ms pulse was reduced during the usAHP (Fig. 5B; paired *t*-test; n = 13, P < 0.01), although the instantaneous firing frequency calculated from the interval between the first and second spikes did not drop (Fig. 5B; paired *t*-test; n = 13, P = 0.18). This indicates that the usAHP introduces a delay to when neurons reach their firing threshold, but once firing is initiated, their frequency is not reduced. This in turn strongly suggests that the membrane hyperpolarization alone cannot explain the observed effects on firing. The same protocol was also applied to rhythmic neurons not displaying a usAHP (see ref. 9 for details). No change in the first spike delay, spike number or firing frequency was observed (Fig. 5B; n = 11, P = 0.46; n = 11, P = 0.84; n = 9, P = 0.37 respectively), ruling out the possibility that the prolonged first spike delay was caused by the long depolarization and repetitive firing used to induce the usAHP.

To confirm that the prolonged first spike delay is caused by the usAHP, 0.5–1 μM ouabain or zero K^+^ saline was applied in the bath to block or arrest sodium pump activity and therefore remove the usAHP. The change in the first spike delay was totally abolished (Fig. 5C,D; n = 6: 4 ouabain [2 cINs; 1 aIN; 1 unknown], 2 zero K^+^ saline [2 MNs]; P < 0.01), suggesting a reliance of the delay on the hyperpolarization due to the usAHP. Next, we wanted to explore the mechanisms involved in the prolonged spike delay.

### Presence of A-type K^+^ current

Our data described above show that the usAHP can modify network excitability by altering non-dIN neuron firing reliability and electrical properties, but the mechanism involved is unknown. The membrane hyperpolarization during the usAHP has no influence on input resistance, but could affect the biophysical state of ion channels, one of which is the A-type K^+^ channel, whose inactivation at rest can be removed by membrane hyperpolarization. A K^+^ current with A-type properties is known to be present in *Xenopus* spinal cord neurons and is involved in regulating their firing^13^,^14^. Recently, it has been shown that non-dINs display delayed firing mediated by a fast transient K^+^ current, which is very likely *I*_*A*_^15^. When the membrane potential is depolarized from more negative membrane potentials, more A-type channels are activated and a larger K^+^ current occurs. However, as it is still unclear whether A-type K^+^ channels are present in *Xenopus* swim neurons, we sought evidence from current and voltage clamp experiments.

**Figure 6:**
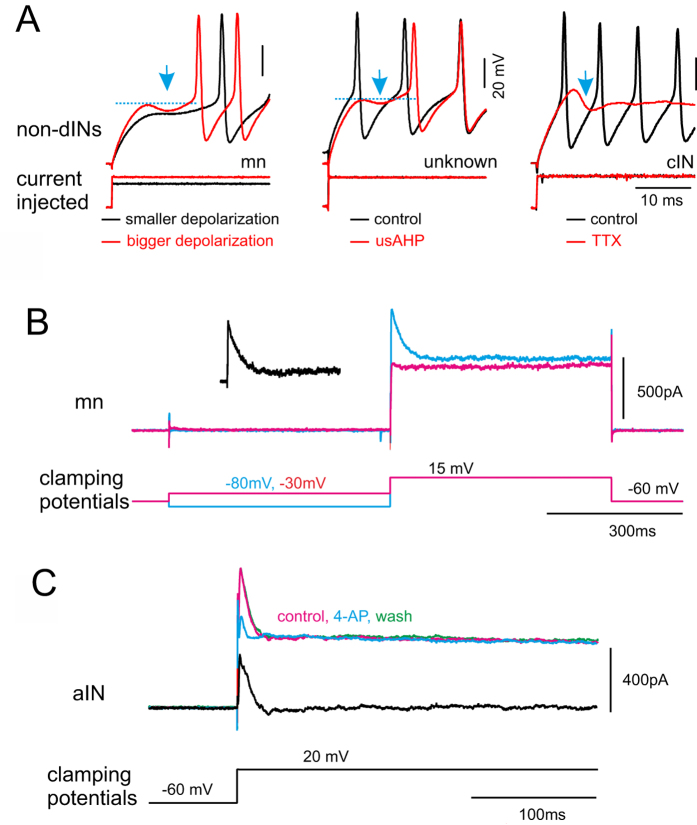
Identifying a voltage-dependent transient outward current. (**A**) In current clamp mode, a small negative membrane potential change (arrow) was occasionally observed during the first spike delay. Left panel shows neuron responses to depolarizing current pulses of two different amplitudes. Black: lower amplitude; red: higher amplitude; the negative membrane potential change appears. Middle panel shows neuron responses to the same depolarizing current pulse during control (black trace) and usAHP periods induced by suprathreshold pulse train (red trace). Right panel shows neuron responses to the same depolarization during control (black trace) and in the presence of TTX (red trace). (**B**) K^+^ currents recorded in voltage clamp mode. Red trace shows a test with pre- clamping potential of −30 mV and blue trace with −80 mV pre-clamping potential. Inset black trace is the difference between the two current traces at the beginning of the voltage step. (**C**) 4-AP at 2 mM preferentially blocks transient K^+^ currents. Red current trace is control, blue is in 4-AP and green is wash. Black trace is the difference in currents between control and 4-AP. (**B**,**C**) are recorded in the presence of 0.4 μM TTX and 200 μM Cd^2+^.

In control conditions, 28 out of 190 recorded rhythmically active swim neurons (14.7%; 4 cINs; 16 MNs; 8 unidentified) displayed a clear negative membrane potential inflection preceding the first spike in a train induced by a depolarising step (Fig. 6A, left panel). This negative inflection suggests that an outward current was activated by the depolarizing pulse. In 3 neurons where a negative membrane potential inflection was absent in control, the inflection was revealed following the induction of a usAHP (Fig. 6A, middle panel). In a further subset of neurons where the inflection was not observed in control, the bath application of 1 μM TTX revealed a similar, small negative membrane potential change (45%; 9 out of 20 neurons; Fig. 6A, right panel), indicating that the outward current was often masked by the first spike. The shape of the negative inflection indicates that this outward current has fast inactivation kinetics, typical of an *I*_*A*_. Therefore, we next wanted to explore whether *Xenopus* CPG neurons, like sensory interneurons and Rohon-Beard neurons^14^, possess a K^+^ current with A-type characteristics. Voltage clamp recordings were made from 11 non-dINs (3 MNs, 4 cINs, 3 aINs and 1 unknown) in the presence of TTX and Cd^2+^ to analyse transient K^+^ currents. In 6 neurons, the pre-holding potential was set at −80 and −30 mV before an activation voltage step to 10–20 mV (Fig. 6B). K^+^ currents evoked from two different holding potentials were subtracted, and a fast activating and inactivating K^+^ current was obtained (Fig. 6B, inset; also see ref. 15 Fig. 1C). Current density was 49 ± 17 pA/pF. In another 5 neurons (3 cINs and 2 aINs), 4-AP (2 mM) was microperfused to test whether the transient K^+^ current is *I*_*A*_^14^. In these experiments, the clamping voltage was stepped from −60 mV to 20 mV. The early K^+^ current peaks were reduced from 822 ± 114 to 622 ± 97 pA (Fig. 6C; P < 0.01) while the late, more steady state K^+^ currents at the end of the 500 ms test pulse were largely unaffected (control: 526 ± 106 pA; 4-AP: 524 ± 100 pA; P = 0.66), confirming the 4-AP sensitive currents are A-type K^+^ currents. These data support the proposal that the increase in spike delay related to the usAHP results directly from the deinactivation of an *I*_*A*_.

### Increasing intracellular Na^+^ by monensin alters CPG neuron firing reliability and network output

**Figure 7:**
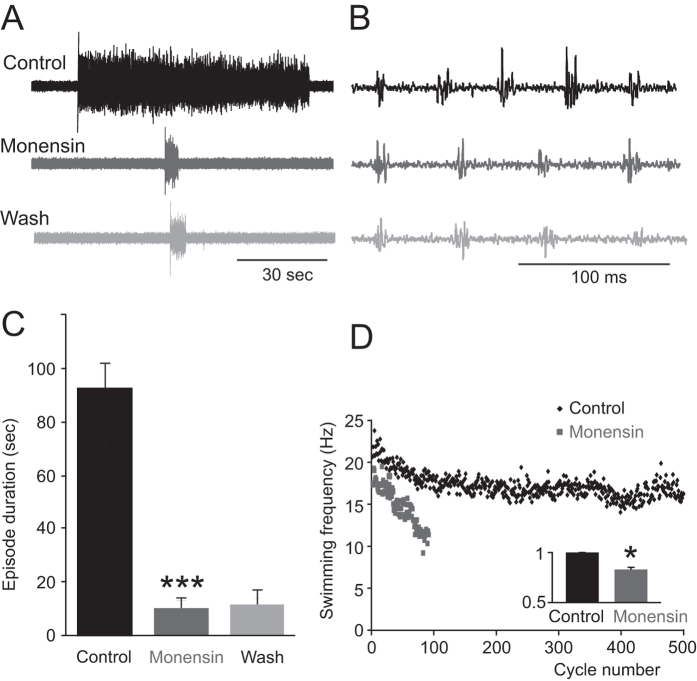
Increasing intracellular Na^+^ concentration using the Na^+^ ionophore monensin shortens swimming episode duration and decreases swimming frequency. (**A**) Bath applying 10 μM monensin irreversibly reduced episode duration. (**B**) Swimming bursts at the beginning of each trace in (**A**). (**C**) Averaged swimming episode durations (***P < 0.001, n = 6). (**D**) Time series measurements showing swimming frequencies of an episode in control and in the presence of monensin. Inset bar graph shows the average normalized swim frequency (*P < 0.05, n = 6).

**Figure 8:**
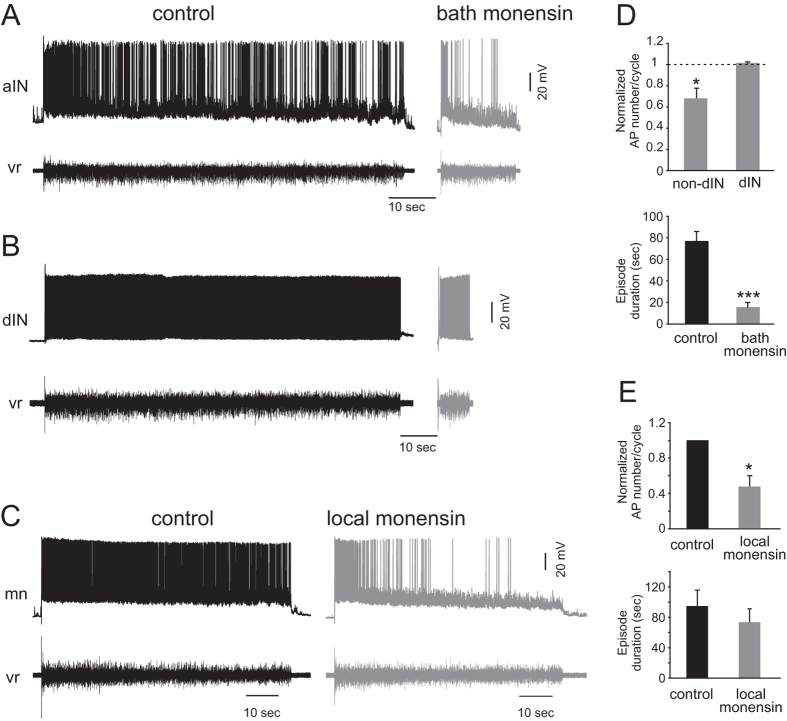
Monensin reduces the firing reliability of non-dINs. (**A**) The firing reliability of non-dINs was reduced by 10 μM monensin. (**B**) dIN firing reliability was not affected. The episode duration was shortened in (**A**,**B**) by adding monensin in the bath. (**C**) Local application of monensin 20 μM directly to the recorded cell, firing reliability of all 5 non-dINs was reduced, whereas the swimming episode duration was almost not affected. (**D**) Averaged normalized firing reliability and episode duration before and after applying monensin in the bath. (**E**) Averaged normalized firing reliability and episode duration in control and during local application of monensin. Black bar: control; grey bar: monensin (*P < 0.05; ***P < 0.001, n = 5).

At the network level, the usAHP can suppress the excitability of the whole motor network. One possible way to enhance a usAHP is to increase pump activity by increasing intracellular Na^+^ concentration. The Na^+^/H^+^ antiporter monensin, which is, in effect, a Na^+^ ionophore, is known to increase intracellular Na^+^ concentration in an electro-neutral manner and subsequently increase the activity of the sodium pump^10^,^11^. Monensin was either applied in the recording bath or locally over the recorded neurons to increase intracellular Na^+^ concentration. Monensin (10 μM; ~10 min) added to the recording bath shortened the swimming episode duration (Fig. 7A,C, n = 6; P < 0.001; Fig. 8D, n = 11; P < 0.001) and slowed down swimming frequency to 82.7 ± 2.3% of control (Fig. 7B,D; P < 0.05), although the effects were not reversible in the time course of the wash period of > 30 minutes. The firing reliability of non-dINs was reduced, but dINs still fired reliably in 10 μM monensin (Fig. 8A,B,D; n = 5; 2 MNs, 2 unknown, 1 aIN; P < 0.05; 6 dINs, P = 0.5). In order to avoid affecting the whole motor control network, monensin (20 μM; ~10 min) was locally applied directly over recorded cells and, as expected, did not shorten swimming episode duration (Fig. 8C,E; n = 5; 3 cINs, 2 MNs). Locally applied monensin reduced the firing reliability of individual non-dIN neurons (Fig. 8C,E; P < 0.05), although without changing their resting membrane potential (see discussion). The effects of monensin, triggered by an increase of intracellular Na^+^ concentration, faithfully mimic the adaptation of the motor network following intense swimming, resulting in a less excitable motor network.

## Discussion

**Figure 9:**
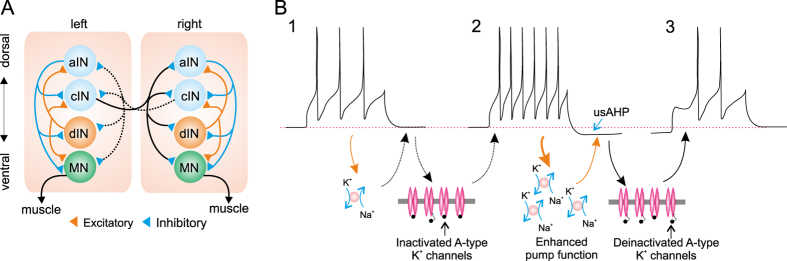
(**A**) Schematic diagram showing the organization of motor circuit in the spinal cord of Xenopus tadpoles at the time of hatching. dINs provide the excitatory drive for swimming and cINs couple the left and right sides. (**B**) Schematic diagram illustrating how the Na^+^ pump and A-type K^+^ current are involved in the short term memory of motor network output. At rest, most A-type K^+^ channels are inactivated. Weak activity (1) does not increase Na^+^ pump current sufficiently to hyperpolarize the membrane potential so when the membrane potential is subsequently depolarized above threshold (2) most A-type K^+^ channels cannot be activated, and thus the first spike delay is unaffected. Stronger activity (2) can potentiate Na^+^ pump function and induce a larger pump current which hyperpolarizes the membrane potential (usAHP). This hyperpolarization removes the inactivation of A-type K^+^ channels. Now, when depolarized above threshold (3), the A-type current is large enough to impede membrane repolarization, prolonging first spike delay, and reducing the total number of spikes.

In this study we have shown an intrinsic self-regulation of spinal motor network function, which reduces the firing reliability of spinal neurons for up to a minute via a usAHP that results from preceding swimming activity, but without affecting rhythm initiation. Rhythm generating capability is altered in terms of cycle frequency, burst intensity and episode duration. The usAHP is caused by the activity-dependent potentiation of Na^+^ pump function^9^, and here we show that it reduces neuronal excitability by shifting the membrane potential away from rheobase and introducing a delay to the first spike. We propose that the deinactivation of *I*_*A*_ by membrane hyperpolarization makes a major contribution to the usAHP mechanism (Fig. 9).

The activity-dependent increase in Na^+^ pump function is triggered by a rise in intracellular sodium concentration. However, other sodium-dependent mechanisms could potentially play a role. For example, Na^+^-activated K^+^ channels are present in *Xenopus* CPG neurons^16^ as in other species (lamprey^4^; rat^17^). However, they are not considered to play a role in regulating CPG neuron firing in the second episode as no K^+^ currents are present during the usAHP period. Furthermore, sodium-independent mechanisms, such as those triggered by extracellular adenosine^18^, could also negatively modulate motor activity. However, this purinergic modulation is unlikely to be involved in the usAHP mechanism as the time course of adenosine production does not match the outcome of our paired episode experiments.

Our previous paper showed a striking linear relationship between swim episode duration and inter-swim interval^9^. In this study, a more systematic analysis of swim parameters was made using pairs of evoked episodes with varying intervals between them, while allowing a rest period of 2 minutes between pairs. When the interval between two episodes is set long enough (> 1 min, longer than the average usAHP duration), the duration and frequency of swimming episodes are very similar (e.g. Fig. 1A,C; Ep 1). However, when set shorter than 30 seconds (e.g. Fig. 1, Ep 2), the interval became very important in determining the subsequent swimming duration and frequency, consistent with our previously published data^9^. Fictive swimming episodes were induced using brief stimulation of the tail skin. Since sensory pathway neurons fire only at the very start (the first ~20 ms) of skin-evoked swimming episodes^19^, they can be ruled out as a factor influencing the second episode of a pair. Instead, we suggest that activity-dependent changes in the CPG neurons themselves are responsible for our observed effects on swim parameters during subsequent network activity. The duration of the first episode also affected the duration of the second episode. When the interval was set to a fixed value (<30 seconds), the longer the first swimming episode, the shorter the second episode (data not shown). These data indicate that the spinal motor network retains a memory not only of when the previous motor activity occurred, but also of how long it lasted. This short term memory has been shown to be mediated by a slow membrane hyperpolarization (usAHP) following swimming^9^, which acts as a spike counting device. The spinal motor network “memorizes” how intense the previous swimming episode was and thus alters subsequent swimming activity accordingly. As the usAHP is mediated by potentiation of Na^+^ pump function^9^, this regulatory mechanism may represent a general intrinsic negative feedback system that is present in many neural networks. Indeed, similar phenomena have been reported in various neuron types, including *Drosophila* motoneurons^6^, mouse pyramidal neurons^8^, leech sensory neurons^5^,^20^, and rat auditory neurons^21^.

Our data show that CPG neuron firing is modulated by the usAHP in a cell type-specific manner. Non-dIN neurons (aINs, cINs and MNs) fire less in the second episode (Fig. 2B), whereas dINs, which provide the excitatory drive for swimming, continue to fire once per cycle (Fig. 2A). Swimming frequency in *Xenopus* tadpoles is determined by the speed of dIN rebound firing, which in turn is affected by mid-cycle inhibition from cINs, as well as dIN tonic depolarization amplitude^12^. Reduced and delayed firing in the cIN population will result in reduced mid-cycle inhibition, subsequently delaying dIN rebound firing, and thus slowing down swimming. The lower and faster decline of swimming frequency in the second episode is therefore very likely due to reduced or delayed cIN firing in the beginning of the second episode, which results in delayed dIN firing and a progressive reduction in swimming frequency. We propose that when too many cINs drop out from the active motor network in the second swim episode, a sufficient number of dINs fail to generate rebound action potentials, causing swimming to terminate prematurely.

This type-specific modulation of CPG neurons is presumably mediated by the usAHP following swimming, which exists only in non-dIN neurons^9^. Here we have shown that the usAHP reduces firing reliability of non-dINs by increasing first spike delay and shifting the membrane potential away from rheobase. No usAHP is seen in dINs, so their firing reliability should not be affected, as confirmed in this study. The preserved firing reliability in dINs due to the lack of effect of a usAHP intuitively will maintain a residual rhythm generating capability, irrespective of inter-swim interval.

In this study we used the Na^+^ ionophore, monensin^10^,^11^,^22^, to increase intracellular Na^+^ concentration and thus mimic the Na^+^ influx into CPG neurons that occurs during swimming, which should subsequently enhance outward Na^+^ pump current to produce a usAHP, at least in a subset of non-dIN neurons. Our data show that application of monensin reduced the firing reliability in non-dINs, but not dINs, similar to the changes in neuronal firing that occur during the weakened second episode (Fig. 2). Bath application of monensin resulted in short swimming episodes with reduced swimming frequency, mimicking the second episode in our earlier experiments (Fig. 1), whereas local application of monensin over individually recorded neurons did not affect the network output. Although monensin, as expected, decreased the excitability of the motor network and individual non-dIN neurons, it did not always induce an obvious usAHP-like membrane hyperpolarization. Therefore, besides the assumed effect of potentiating Na^+^ pump function, additional effects may also be triggered by monensin, including changes in membrane conductance^23^ and synaptic strength^24^,^25^. However, more recent studies in neurons^11^ suggest that the effects are primarily due to the Na^+^ pump and not to changes in synaptic transmission. Furthermore our own data also do not support a change in membrane conductance in the presence of monensin (data not shown).

Transient *I*_*A*_ currents play an important role in regulating the action potential repolarizing phase, firing frequency and onset of action potentials in many neuron types^26^,^27^,^28^,^29^,^30^. Studies in *Drosophila* using a Shal/K_v_4 knockout, which encodes *I*_*A*_ in neuronal cell bodies, have shown that *I*_*A*_ is required for the initiation of firing, repetitive firing and the generation of rhythmic motor behaviors such as crawling, climbing and grooming^31^. In lamprey, both motoneurons and crossed caudally projecting interneurons display a high-voltage-activated *I*_*A*_, which plays a role in spike timing and locomotor pattern generation^32^. An *I*_*A*_ has also been reported in isolated *Xenopus* embryo spinal neurons^13^. *In vivo* experiments have shown that *Xenopus* Rohon-Beard neurons and sensory interneurons display an *I*_*A*_-like current, which could be involved in regulating their firing^14^. More recently it has been shown that *Xenopus* non-dINs possess an *I*_*A*_, and it can increase firing threshold^15^. In this study, we have shown that *I*_*A*_ very likely plays a significant role in regulating spike onset timing, but not subsequent firing frequency. As channels mediating the *I*_*A*_are partially inactivated at rest and can be de-inactivated at more negative membrane potentials (Fig. 6B), a larger *I*_*A*_ can be evoked during the usAHP. In turn, this outward current will prevent or postpone non-dIN firing induced by depolarizing pulses (Fig. 9) or EPSPs during swimming. Therefore, reduced firing reliability and delayed firing will result in slower and shorter swimming episodes.

The *I*_*A*_ was only occasionally seen in current clamp mode, perhaps because it is masked by fast Na^+^ currents contributing to the rising phase of action potentials. After blocking fast Na^+^ currents with TTX, a rapidly inactivating outward current, presumably an *I*_*A*_, was seen more often. The majority of neurons displaying this *I*_*A*_-like current were cINs and MNs. It has been shown that cINs and MNs display a longer spike delay compared to dINs upon receiving each EPSP, and cINs also display a delay between a single initial spike and a subsequent burst when depolarized by suprathreshold current injection^33^. Therefore we assume that the long spike delay of these two types of neurons is caused by an *I*_*A*_.

Our data support the hypothesis that the Na^+^ pump-mediated short term memory displayed by the *Xenopus* tadpole swim motor network^9^ is mediated by the deinactivation of *I*_*A*_ due to the usAHP and membrane potential hyperpolarization. This short term memory decreases and delays CPG neuron firing and consequently slows down swimming. As Na^+^ pumps are ubiquitously distributed and *I*_*A*_ has been shown in many neuron types, this mechanism likely represents a broadly conserved negative feedback regulation of neural networks generating rhythmic activity.

## Materials and Methods

### Experimental animals

All experiments were performed on newly hatched *Xenopus laevis* tadpoles at developmental stage 37/38 or 42^34^. Tadpoles were reared from fertilized ova obtained following breeding of adults selected from an in-house colony. Mating was induced by injections of human chorionic gonadotropin (HCG) into the dorsal lymph sac of breeding pairs of adult frogs. All experiments were approved by the Animal Welfare Ethics Committee (AWEC) of the University of St Andrews and conformed to UK Home Office regulations.

### Electrophysiology

*Xenopus* tadpoles were immobilized in 12.5 μM α-bungarotoxin saline after their trunk skin had been gashed, and mounted on a rotatable Sylgard platform in a bath of saline (in mM: 115 NaCl, 2.5 KCl, 2 CaCl_2_, 2.4 NaHCO_3_, 1 MgCl_2_, 10 HEPES, adjusted with 4 M NaOH to pH 7.4). One or both sides of the trunk skin overlying the myotomal muscles were removed. The dorsal parts of rostral myotomes were freed from the spinal cord and the roof of the hindbrain was opened to the neurocoel to improve drug access and access for patch clamp electrodes. Exposed neuronal somata were patch clamped using borosilicate glass pipettes (Harvard Apparatus Ltd) pulled on a Sutter P97 pipette puller. Patch pipettes were filled with 0.1% neurobiotin (Vector lab) in the intracellular solution (in mM: 100 K-gluconate, 2 MgCl_2_, 10 EGTA, 10 HEPES, 3 Na_2_ATP, 0.5 NaGTP adjusted to pH 7.3 with KOH) and had resistances of ~10 MΩ. Changes in membrane potential were recorded in current clamp mode using an Axoclamp 2B amplifier. Simultaneous extracellular recordings of fictive swimming were made with suction electrodes from ventral roots at intermyotomal clefts, and signals were amplified using differential AC amplifiers (A-M Systems Model 1700). Simultaneous intracellular and extracellular signals were digitized using a CED Power 1401, and displayed and stored on a PC computer using Spike 2 software. Microperfusion of monensin was applied using a pipette (~10 μm in diameter) positioned close to the recorded soma. Voltage clamp recordings were made using Multiclamp 700 B. Series resistance (20–50 MΩ) was compensated by 70–80%, and recordings with series resistance over 50 MΩ were not used for analysis. The uncompensated series resistance would not cause big errors in actual clamping potentials when the currents were small but will produce significant errors at more depolarised levels. Any errors in clamping potential were manually corrected offline by an amount determined by multiplying peak potassium currents by 20–30% of series resistance. The junction potential against standard saline, calculated using Clampex 10.2 junction potential formula, was 14.7 mV. This was not corrected in the analyses. Leak currents were all subtracted during experiments. To isolate potassium currents, 0.4 μM TTX and 200 μM Cd^2+^ were included in the saline^16^. Microperfusion of 4-aminopyridine (4-AP) was used to block transient potassium currents using a pipette (~10 μm in diameter) positioned close to the recorded soma. Fictive swimming was initiated either by dimming the illumination or by stimulating through a glass suction electrode placed on the tail skin^35^, which delivered a 1 ms current pulse via a DS2A isolated stimulator (Digitimer).

### Data analysis

Electrophysiological data were first analysed using Dataview software (v 8.3, courtesy of Dr. W. J. Heitler) and all raw data were imported into Excel spread sheets and analysed. Statistical analyses were conducted using PASW statistics 21 or Excel. Pairs of evoked episodes obtained for each interval condition (5, 15 and 30 seconds) were always given a rest period of two minutes between each pair. For each pair of episodes, the frequency of each swim cycle in episode 2 was normalised to the equivalent cycle in episode 1 and normalised values were compared using a Wilcoxon signed rank test (c.f. ref. 12). Swim frequency during monensin experiments was also analysed using the same method as in the interval experiments. Firing reliability of CPG neurons was calculated by counting action potential number of either the first 30 swim cycles or all cycles of each swimming episode, and dividing by the number of cycles. Means of each condition were compared using paired two-tailed *t*-test or one-sample t-test, and shown as mean ± SEM.

### Neuron anatomy

Following patch recordings, animals were fixed in 2% glutaraldehyde in 0.1 M phosphate buffer, pH 7.2, overnight in a refrigerator (∼4 °C). Animals were first rinsed with 0.1 M PBS (120 mM NaCl in 0.1 M phosphate buffer, pH 7.2), and washed in two changes of 1% Triton X-100 in PBS for 15 min with agitation. Next, animals were incubated in a 1:300 dilution of extravidin peroxidase conjugate in PBS containing 0.5% Triton X-100 for 2–3 hours with agitation, and washed again in at least four changes of PBS. Animals were then immersed in 0.08% diaminobenzidine in 0.1 M PBS (DAB solution) for 5 min, moved to a DAB solution with 0.075% hydrogen peroxide for 1–2 min, and then washed in running tap water. Finally, animals were dehydrated in 100% alcohol, cleared in methyl benzoate and xylene, and mounted whole, between two coverslips using Depex. Neuronal cell bodies and axons were observed under a X40 objective to identify CPG neuron types. All reagents were obtained from Sigma or Tocris Bioscience.

## Additional Information

**How to cite this article**: Zhang, H.-Y. *et al.* Mechanisms underlying the activity-dependent regulation of locomotor network performance by the Na^+^ pump. *Sci. Rep.* **5**, 16188; doi: 10.1038/srep16188 (2015).
