# Corrigendum: Mechanisms underlying the activity-dependent regulation of locomotor network performance by the Na^+^ pump

**DOI:** 10.1038/srep46909

**Published:** 2017-12-22

**Authors:** Hong-Yan Zhang, Laurence Picton, Wen-Chang Li, Keith T. Sillar

Scientific Reports 5: Article number: 16188; 10.1038/srep16188 published online: 11 06 2015; updated: 12 22 2017.

In this Article, ‘Centre for Neuroregeneration, University of Edinburgh, Chancellor’s Building, 49 Little France Crescent, Edinburgh, EH16 4SB’ is incorrectly listed as a present address for Hong-Yan Zhang and should be listed as a second affiliation.

